# Modafinil Ameliorated Fibromyalgia Syndrome in Rats by Modulating Mast Cells and Microglia Activation Through Dopamine/Substance P/MRGPRX/Histamine and PI3K/p-Akt/NF-κB Signaling Pathways

**DOI:** 10.1007/s11481-025-10194-6

**Published:** 2025-04-15

**Authors:** Mennat-Allah M. Kamal, Reham M. Essam, Noha F. Abdelkader, Hala F. Zaki

**Affiliations:** 1 Central Health Laboratories, Ministry of Health, Cairo, Egypt; 2 Department of Pharmacology, Faculty of Pharmacy, Cairo University, Cairo, Egypt; 3 Biology Department, School of Pharmacy, Newgiza University, Giza, Egypt

**Keywords:** Fibromyalgia, Mast cell/Microglia, Modafinil, Reserpine, DA/SP/MRGPRX/Histamine, PI3K/p-Akt/NF-κB, Thalamus

## Abstract

Fibromyalgia syndrome (FMS) is characterized by prolonged, widespread musculoskeletal pain accompanied by various physical and psychological disturbances. Modafinil, a wake-promoting drug, manages pain symptoms in several diseases by inhibiting dopamine reuptake and exhibiting anti-inflammatory and immunomodulatory effects, including the impairment of cytokine production, microglia, and mast cell activation. Central inflammation may involve microglial activation, which is correlated with mast cell activation. Restoring dopamine levels and modulating the communication between mast cells and microglia may represent a promising approach to managing pain symptoms in FMS. Thus, this study intended to explore the interplay between brain mast cells and microglia as an underlying mechanism in the pathophysiology of FMS and how this interaction is controlled by modafinil, with a focus on dopamine/SP/MRGPRX2/histamine and PI3K/p-Akt/NF-κB signaling pathways. Rats were arbitrarily distributed between 4 groups. Group 1 served as normal control. Reserpine (1 mg/kg/day; s.c) was injected into the remaining groups for three consecutive days. In groups 3 and 4, modafinil (100 mg/kg/day; p.o) was administered either alone or in conjunction with haloperidol (1 mg/kg/day; ip), respectively, for the following 21 days. Modafinil ameliorated reserpine-induced thermal/mechanical allodynia (1.3-fold, 2.3-fold) and hyperalgesia (0.5-fold), attenuated depression (0.5-fold), and enhanced motor coordination (1.2-fold). It mitigated the histopathological alterations and increased dopamine levels in the thalamus of rats by 88.5%. Modafinil displayed anti-inflammatory effects via inhibiting mast cells and microglia activation, manifested by reductions in SP/MRGPRX2/IL-17/histamine (52%, 58%, 56.7%, and 63.7%) and PI3K/p-Akt/t-Akt/NF-κB/TNF-α/IL-6 (31.7%, 55.5%, 41%, 47.6%, and 76.9%), respectively. Ultimately, modafinil alleviated FMS behavioral, histopathological, and biochemical abnormalities and suppressed mast cell-microglial neuroinflammation in the thalamus of rats exposed to reserpine. This study highlights the potential of repurposing modafinil to improve FMS symptoms.

## Highlights

• Mast cells-microglial communication contributes to the pathogenesis of FMS.

• Modafinil halted the progression of reserpine-induced FMS in rats.

• Modafinil alleviated mast cell-microglia activation cascades.

• Modafinil suppressed SP/MRGPRX/Histamine and PI3K/p-Akt/NF-κB signaling.

**Graphical Abstract:**

Mast cell-microglial activation cascade in the thalamus: Persistent pain and neuroinflammation reduce DA and induce IL-17, which triggers mast cell activation through SP and MRGPRX. Histamine is one of the mediators secreted, activating microglia via PI3K/AKT/NF-κB. This activation of microglia results in the release of multiple inflammatory mediators, including TNF-α and IL-6, which degrade DAergic neurons. DA, Dopamine; SP, Substance P; IL, interleukins; PI3K, phosphotylinosital 3 kinase; p-Akt, phosphorylated protein serine-threonine kinase; NF-κB, nuclear factor-kappa B; TNF-α, Tumor Necrosis Factor Alpha; MRGPRX, Rat MAS Related GPR Family Member G.

## Graphical abstract

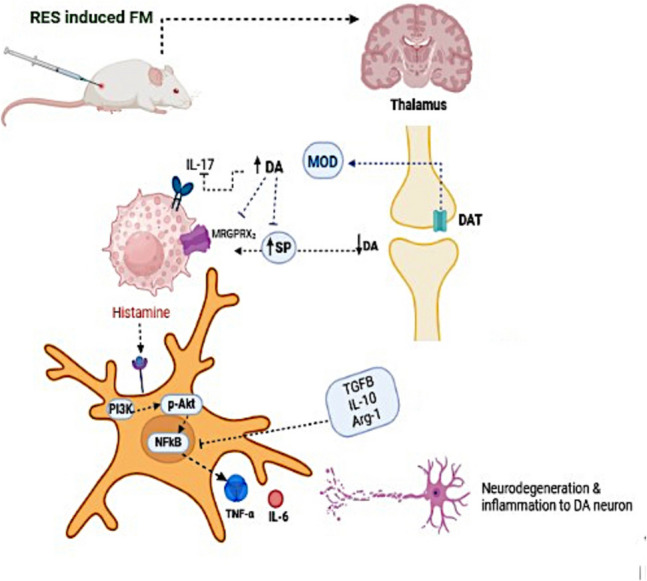

## Introduction

Fibromyalgia syndrome (FMS) is a condition characterized by multi-focal pain, affecting about 2–4% of the population, with 80–90% of patients being female (Wolfe et al. 2018; Sarzi-Puttini et al. 2020). This condition is regarded as the third most predominant musculoskeletal disorder, drastically increasing with age (Sarzi-Puttini et al. 2020). The disease involves different symptoms, such as fatigue, allodynia, hyperalgesia, irritable bowel, cognitive dysfunction, depression, and sleep disorders, without a clearly recognized biological etiology (Arnold et al. 2019; Sarzi-Puttini et al. 2020). However, it can be linked to specific conditions including neurological, mental, or rheumatic disorders (Bellato et al. 2012; Chinn et al. 2016).

The pathogenesis of FMS remains poorly comprehended, and its symptoms are non-specific, overlapping with other disorders (Schmidt-Wilcke and Clauw 2011; Bains et al. 2023). The spinothalamic pathway is widely recognized as the primary ascending pathway for the transmission of pain signals (You et al. 2022). Additionally, evidence suggests that glial cells may contribute to central sensitization and neuroinflammation by generating interleukins (ILs), which are elevated in patients with FMS (Rodriguez-Pintó et al. 2014), as well as enhancing the pain experience even in the absence of the initial painful stimulus (Vandenbark et al. 2019). Different inflammatory mediators, such as substance P (SP), brain-derived neurotrophic factor (BDNF), and glutamate, activate glial cells. The activated glial cells then release pro-inflammatory cytokines, like IL-17, which induce neuroinflammation and lead to chronic pain, fatigue, hyperalgesia, and allodynia in FMS (Albrecht et al. 2019). There is mounting evidence that the decline of dopamine (DA) function is one of several neurological changes observed in chronic pain, particularly FMS. Moreover, a hypodopaminergic status and reduced availability of DA receptors have been linked to increased pain sensitivity (Ledermann et al. 2016). Microglia express several dopaminergic receptors, especially D2 receptors, which regulate neuroinflammation and cell survival (Dominguez-Meijide et al. 2017). It has been proposed that microglial D2 receptor stimulation reduces neuroinflammation (Zhang et al. 2015).

Interestingly, mast cells have been recognized as potential inflammatory mediators in many diseases. They are located in the thalamus, hypothalamus, and median eminence (Grigorev and Korzhevskii 2021). These cells are activated during chronic pain and neuroinflammation via the action of SP on the mast cell receptor-MAS-related G protein-coupled receptor-X2 (MRGPRX2) (Che et al. 2018). This interaction may cause the secretion of several mediators, such as histamine and tryptase, which then activate microglia, aggravating pain sensation and entering a viscous loop (Dong et al. 2014; Zhang et al. 2016). Hence, studying the imprint of mast cells degranulation on microglia activation and its relevance to the pathogenesis of FMS seemed appealing.

Developing an animal model that mimics FMS is a critical step in establishing therapeutic approaches for preclinical research (Brum et al. 2022). Repeated subcutaneous administration of reserpine (RES) is a well-established model in rats that mimics the key features of FMS in humans, such as chronic widespread pain and common comorbidities, like depression, as a result of the depletion of central biogenic amines (Nagakura 2022).

Both pharmacological and non-pharmacological therapeutic methods are frequently required for the management of FMS (Bair and Krebs 2020). There is no definitive cure for FMS; only medications are available to control the symptoms (Oliveira Júnior and Almeida 2018). Only pregabalin, duloxetine, and milnacipran have received FDA approval to manage FMS (Tzadok and Ablin 2020). Other pharmacological classes, including N-methyl-D-aspartate receptor antagonists, DA agonists, and dopamine reuptake inhibitors, can be promising in FMS (Lawson 2017).

Modafinil (MOD) is a non-amphetamine stimulant, which promotes wakefulness. MOD's exact mechanism of action is elusive, although the primary action of MOD is mostly by enhancing catecholamine neurotransmission (Minzenberg et al. 2016). Modafinil directly elevates dopamine and norepinephrine levels by inhibiting their uptake transporters, which subsequently boosts glutamate, orexin, serotonin, and histamine levels while indirectly reducing gamma-aminobutyric acid level (Battleday and Brem 2015). Modafinil is an FDA-approved drug for the management of sleep disorders, such as narcolepsy, sleep apnea, and excessive daytime sleepiness. However, it is extensively used off-prescription to treat fatigue in many neurological and neurodegenerative disorders, in addition to cognitive enhancement (Battleday and Brem 2015; Zager 2020; Pliszka 2022). It possesses a satisfactory safety profile and a low abuse liability (Pliszka 2022). In 2007, a simple retrospective study revealed that MOD may effectively ameliorate fatigue associated with fibromyalgia (Schwartz et al. 2007). However, no study has explored its neuroprotective potential in FMS. Noteworthy, MOD has recently shown potent neural immunomodulatory and anti-inflammatory activities (Zager et al. 2018; Zager 2020; Seadawy et al. 2020; Ghorbanzadeh et al. 2022; Bagcioglu et al. 2023). The administration of MOD reduced motor impairment and anxiety- and depressive-like behaviors in the mice model of lipopolysaccharide (LPS)-induced sickness and depressive-like behaviors (Zager et al. 2018). Modafinil mitigated the key features of rotenone-induced Parkinson’s disease in rats via embedding oxidative stress and neuroinflammation (Seadawy et al. 2020). Modafinil exerted analgesic and neuroprotective effects against sciatic nerve cuffing-induced neuropathic pain in mice, possibly via its anti-inflammatory and nitrergic/serotonergic modulatory abilities (Ghorbanzadeh et al. 2022). Furthermore, MOD improved autism‑like behavior and neuroinflammation in the propionic acid rat model (Bagcioglu et al. 2023). The dysfunction of the dopaminergic system is associated with enhanced pain sensation and impaired mental status (Ledermann et al. 2021). Therefore, strategies for restoring DA signaling and understanding the connection between mast cells and microglia may represent a promising approach to managing pain symptoms in FMS. To summarize, it was hypothesized that MOD could alleviate FMS by enhancing dopaminergic activity and modulating mast cell/microglial activation. The current investigation evaluated the effects of MOD on the RES-induced FMS model in rats with a focus on DA/SP/MRGPRX/histamine/neuroinflammation signaling, using haloperidol (HAL) (a DA receptor antagonist).

## Materials and Methods

### Animals

Forty male Wistar rats, weighing 200–220 g, were acquired from the Modern Veterinary Office for Laboratory Animals, Giza, Egypt. Male rats were utilized in this study to examine the effects of MOD without being influenced by the female rats' hormones or estrous cycle, especially that male and female rats exhibit FMS following RES administration in a comparable manner (Nagakura et al. 2009). Rats were kept under controlled room temperature, humidity, and a light/dark cycle of 12/12 h. They were allowed free access to food and water. The investigation complies with the Guide for Care and Use of Laboratory Animals issued by the US National Institutes of Health (NIH Publication No. 85–23, revised 2011), following the ARRIVE guidelines. All the experiment steps were approved by the Ethics Committee for Animal Experimentation at the Faculty of Pharmacy, Cairo University, Egypt (PT: 2823).

### Drugs and Chemicals

Reserpine, MOD, and HAL were purchased from Sigma-Aldrich (St. Louis, MO, USA). Reserpine was dissolved in glacial acetic acid and diluted to a final concentration of 1 mg/mL of 0.5% acetic acid with distilled water. Modafinil and HAL were dissolved in saline. Other chemicals used in this study were of high analytical grade.

### Reserpine Administration

To induce FMS, each rat was injected subcutaneously with RES (1 mg/kg) daily for three consecutive days to deplet the biogenic amines (Nagakura et al. 2009).

### Experimental Design

**Fig. 1:**
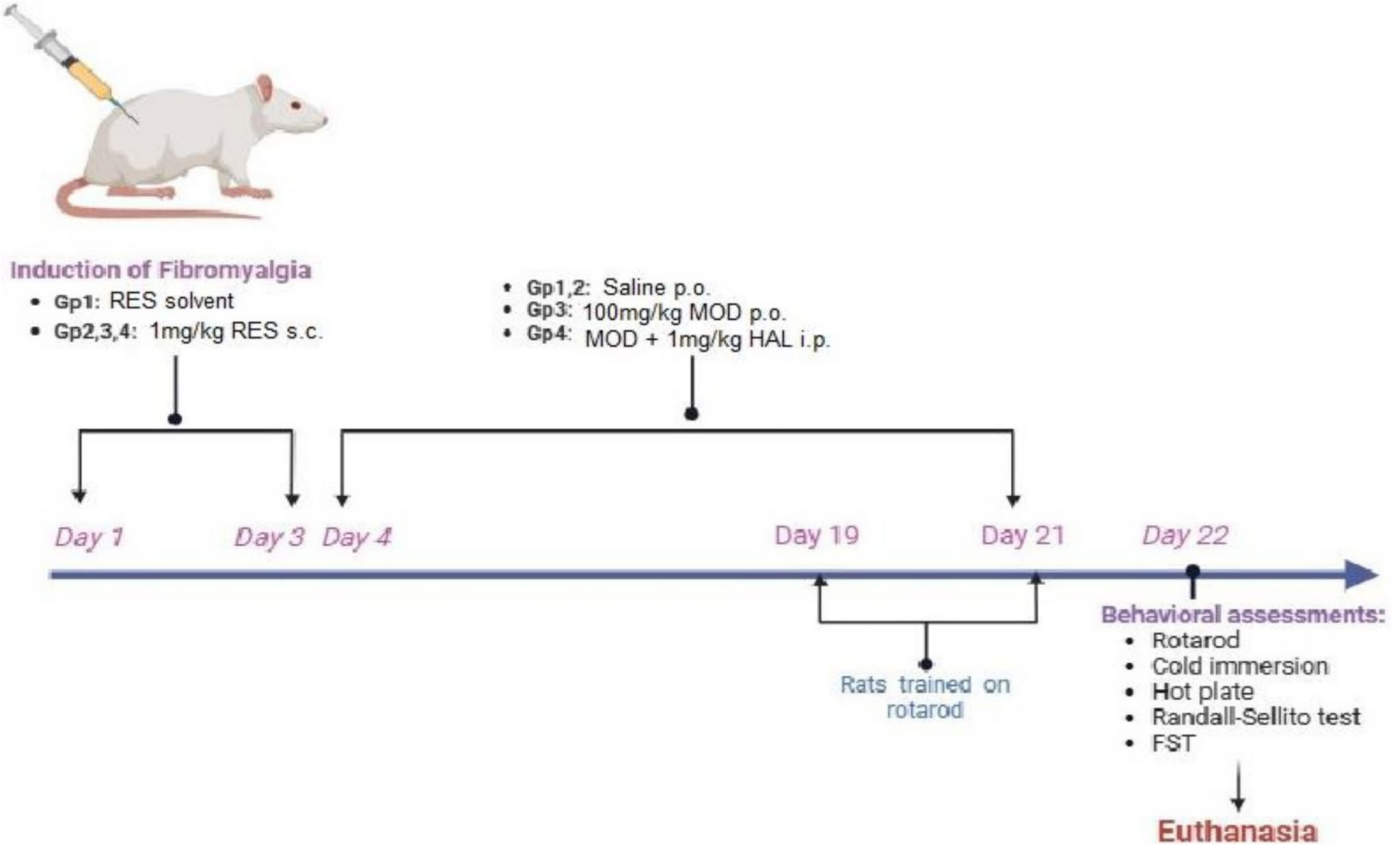
Illustration of the experimental design timeline. RES: reserpine, MOD: modafinil, HAL: haloperidol

As shown in Fig. 1, rats were arbitrarily allocated to four groups (*n* = 10). Group I (Normal): rats received 0.5% glacial acetic acid subcutaneously and represented the control group. Group II (RES): rats received RES (1 mg/kg, s.c) once daily for three consecutive days and represented the FMS group. Following three days of RES injection, rats of group III (RES + MOD) received MOD (100 mg/kg, p.o.) (Moreira et al. 2010), while those of group IV (RES + MOD + HAL) received HAL (1 mg/kg, i.p.) (Vasconcelos et al. 2003) before the administration of MOD, starting on day four and continued for 21 days. Rats were trained daily in the last three days of the experiment on an automated five-lane rotarod. At the termination point of the experiment, motor coordination was assessed using the rotarod test, while thermal allodynia/hyperalgesia and mechanical hyperalgesia were monitored through cold immersion, hot plate, and Randall-Sellito tests, respectively. Furthermore, the influence of FMS on mood quality was tested by a forced swimming test (FST). The order of the behavior tests commenced with the least stressful test and concluded with the most stressful one. Moreover, equipment were sanitized using 70% ethanol after each rat to remove any animal cues. After behavioral testing, the animals were sacrificed under light anesthesia using thiopental sodium (50 mg/kg, i.p.) (Gazdhar et al. 2013), and their brains were quickly excised, washed with ice-cold saline, and divided into two sets. The first set (*n* = 3) was subjected to immediate fixation in 10% formalin for 72 h to perform histopathological staining with hematoxylin/eosin (H&E) and toluidine blue along with immunohistochemical analysis of activated microglia using ionized calcium-binding adaptor molecule 1 (Iba-1) stain. The second set was weighed, and the thalami were collected. Parts of three different thalami were used for western blotting. The rest of the second set was homogenized at 3000 × g (4 °C for five minutes) in ice-cold saline to prepare 10% homogenates, then centrifuged at 10,000 × g for twenty minutes to obtain the supernatants, which were stored at −80 °C for biochemical assessment. Carcasses were frozen at −80 ºC till incineration. Throughout the experimental work, all samples were kept anonymous, and an independent investigator coded and decoded them.

### Behavioral Tests

#### Rotarod Performance Assessment

In this procedure, rats were placed on the automated five-lane rotarod apparatus (Model 47750, Ugo Basile, Italy) with a fixed speed of 14 rpm for five minutes. Training sessions were conducted three days before testing (one session per day). The duration of the rat’s descent off the rotating rod throughout the five-minute interval was recorded (Murai et al. 2017).

#### Cold Immersion Test

The distal portion of the tail (5 cm) was submerged in a container of cold water at 4 ± 1 °C. The duration of time each rat took to withdraw its tail from cold water was recorded. A cut-off time of 15 s was selected to avoid tissue injury (Jung et al. 2017).

#### Hot Plate Test

Rats were positioned separately on a hot plate (Model 7280, Ugo Basile, Italy), where its temperature was adjusted at 55 ± 1 °C. The response latency was recorded as the time the rat takes to shake, jump off the surface, or lick the hind paw. A cut-off time of 20 s was selected to prevent tissue injury (Kamel et al. 2022).

#### Randall-Selitto Test

Randall-Selitto assay measures withdrawal reactions caused by applying a mechanical force that gradually increases to the mid-gastrocnemius muscle of the rat’s hind paw. (Model 7200, Ugo Basile, Italy). A pointed cylindrical mechanical probe was used to apply progressively growing pressure to the rat’s hind paw’s dorsum. Rats were gently restrained, and mechanical pressure was raised till vocalization or a withdrawal response manifested. The thresholds were measured in grams. An arbitrary cut-off value of 250 g was applied (Abdelkader et al. 2022).

#### Forced Swimming Test

As previously described, mood disturbance was assessed using FST (Atta et al. 2023a). In this procedure, rats were placed in a cylindrical plastic tank filled with water that was kept at a height of 17 cm and a temperature of 23 ± 2 °C. The immobility time during the five-minute period was recorded. Immobility time is the absence of all movements except those needed to keep the head above the water surface.

### Biochemical Parameters

#### ELISA Analysis

Rat-specific ELISA kits obtained from ELK Biotechnology (CO, USA) were used to measure the thalamic levels of DA (Cat. No. ELK8953) and PI3K (Cat. No. ELK8431). However, thalamic levels of histamine (Cat. No. MBS732202), IL-17 (Cat. No. MBS2022678), SP (Cat. No. MBS703659), and TNF-α (Cat. No. MBS2507393) were estimated by rat-specific ELISA Kits purchased from MyBioSource (CA, USA). Moreover, IL-6 (Cat. No. SEA079Ra, Cloud-Clone Corp, Wuhan, China), NF-κB (Cat. No. CSB-E13148r, CUSABIO, Wuhan, China), and MRGPRX (Cat. No. abx533638, Abbexa Ltd, Texas, USA) were also analyzed by ELISA technique. All these tests were assessed following the manufacturer’s instructions supplied with each kit.

#### Western Blotting

Samples from the thalamus were centrifuged for 15 min at 4 °C after being mechanically homogenized in a buffer containing protease inhibitors (buffer constituents: 0.16 mol/L of NaCl, 0.11 mol/L of Tris–HCl “pH 8.0”, 2.1 mol/L of EDTA “pH 8.0”, 0.01% of Triton, and 9.04 mg/mL of diethylpyrocarbonate; Sigma, St. Louis, MO). Samples were added to a vertical 10% SDS-PAGE (20 μg protein/well). The proteins were eliminated by applying 150 V to the gel and then transferred to a nitrocellulose membrane (Hyperbond-Enhanced Chemiluminescence, Amersham Pharmacia Biotech, Buckinghamshire, UK). For one hour, membranes for p-Akt were blocked with 7% nonfat dry milk in Tris HCl-buffered saline containing 0.1% Tween 20 (TBST), while membranes for T-Akt were blocked with 5% nonfat dry milk in TBST. Primary antibodies targeting T-Akt (polyclonal anti-Akt1 rabbit IgG; Bio-Rad laboratories, CA, USA; 1:2,000 in TBST with 5% nonfat dry milk) and p-Akt (polyclonal anti-phospho-Akt (Ser473) rabbit IgG; New England Biolabs, MA, USA; 1:1,000 in TBST) were incubated on the membranes overnight at 4 °C. Thereafter, membranes were incubated for one hour, membranes were incubated with either horseradish peroxidase-conjugated secondary antibody for p-Akt or T-Akt (Amersham Pharmacia Biotech). Membranes were developed using enhanced chemiluminescence and subjected to Hyperfilm in accordance with Amersham Pharmacia Biotech’s manufacturer’s instructions. The membranes were chemically stripped of antibodies before being reprobed, using a stripping buffer (2% SDS, 20 mmol/L of Tris “pH 6.5,” and 7 mmol/L of 2-mercaptoethanol). The chemiluminescent substrate (ClarityTM Western ECL substrate Bio-Rad, Cat. No. 170–5060) was added to the blot, and the resulting chemiluminescent signals were captured by a CCD camera-based imager. The intensity of the bands corresponding to the target proteins was evaluated using image analysis software, with normalization against β-actin.

### Histopathological Examination

The whole brains of three animals per group were excised and fixed in 10% neutral-buffered formalin. After 72 h, brain samples were processed using serial grades of ethanol and then cleared in xylene. Samples were infiltrated and embedded using Paraplast embedding media. Later, samples were sectioned to a thickness of five μm and stained with H&E to investigate structural abnormalities and with toluidine blue as an indicator of mast cell activation. An independent investigator performed the histopathological and immunohistochemical analysis blindly to obliterate any possible bias and ensure the results' transparency.

### Immunohistochemical Analysis

Microglial activation was measured immunohistochemically using Iba-1 staining. Brain Sects. (4–5 μm) were sliced on adhesive slides, deparaffinized, and retrieved. The brain samples were incubated overnight at 4 ℃ with anti-Iba-1 primary antibody (Cat. No. MA5-27726, ThermoFisher Scientific, Waltham, USA) at a dilution factor of 1:1000. Then, a two-hour incubation with HRP-labeled secondary antibody (Abcam, Cambridge, UK) was done, followed by the addition of DAB-substrate for detection. The positive reaction was measured as the area percentage of expression in five randomly selected, non-overlapping fields from each sample, analyzed by CellSens dimensions (Olympus software).

### Statistical Analysis

Data was expressed as mean ± SD. Statistical analysis was performed using a one-way ANOVA test followed by Tukey’s as a post hoc test for multiple comparisons using GraphPad Prism statistical software (version 9.00 for Windows, San Diego, CA, USA). For all statistical testing, the significance level was set at *p* < 0.05.

## Results

### Modafinil Diminished Allodynia and Hyperalgesia in Reserpine-induced Fibromyalgia in Rats

**Fig. 2:**
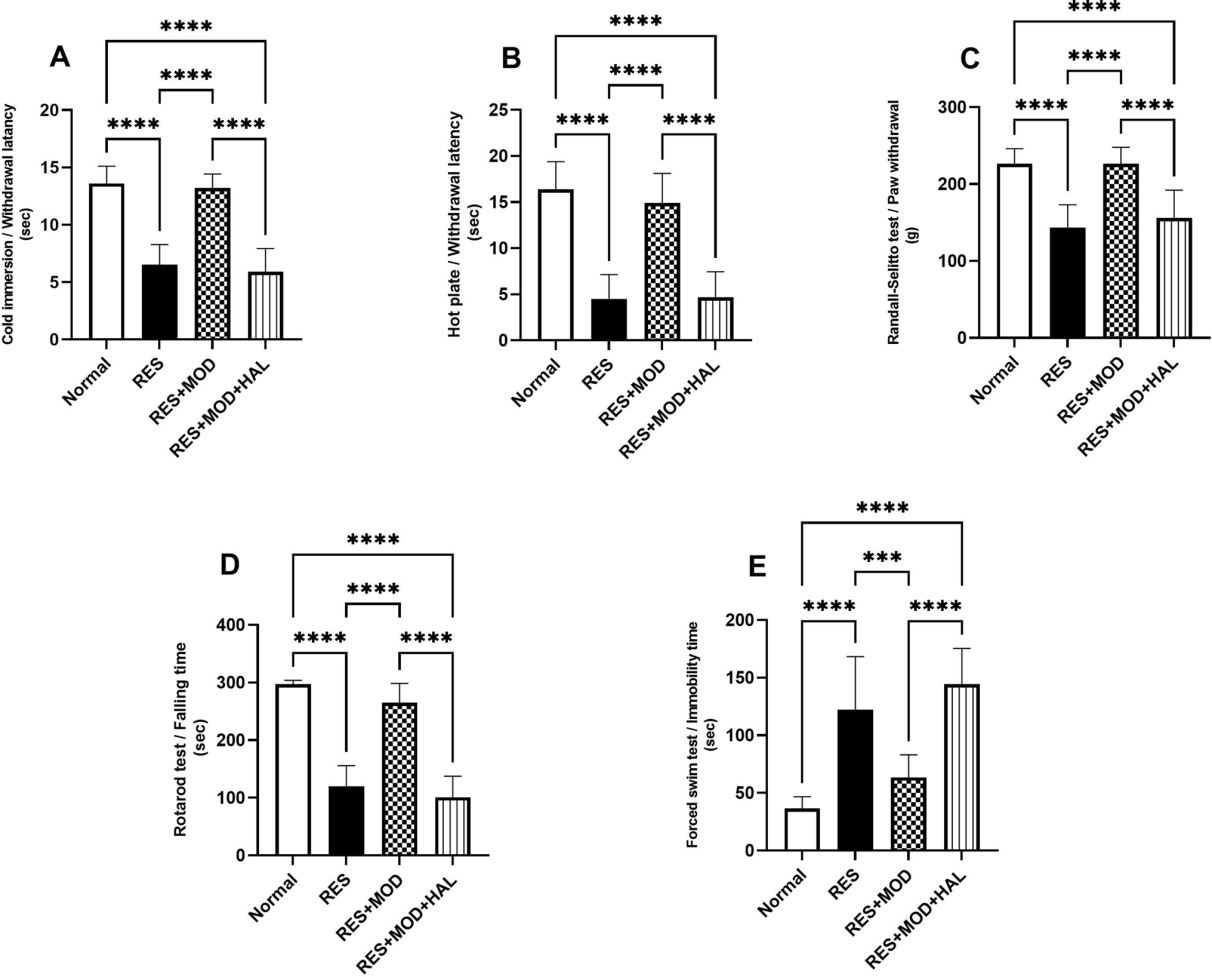
Effect of modafinil alone and combined with haloperidol on reserpine-induced allodynia, hyperalgesia, motor coordination, and depression using cold immersion test (**A**), hot plate test (**B**), Randall-Selitto test (**C**), rotarod test (**D**), and forced swim test (**E**). Each bar with a vertical line represents mean ± SD (*n* = 10) using a one-way ANOVA test followed by Tukey’s as a post hoc test for multiple comparisons with a value *p < 0.05, **P < 0.01, ***P < 0.001, and ****P < 0.0001

Subcutaneous RES administration caused a significant increase in allodynia and hyperalgesia, evidenced by a decrease in tail withdrawal latency in the cold immersion test, hot plate-paw withdrawal latency, as well as Randall-Selitto-paw withdrawal threshold by 52.2, 72.56, and 36.6%, respectively, when compared to the normal rats. The administration of MOD reversed the effects of RES, causing a marked decrease in hyperalgesia and allodynia. Modafinil significantly increased tail withdrawal latency, paw withdrawal latency, and paw withdrawal threshold by 1.3-, 2.3-, and 0.5-fold, respectively, as compared to the RES group. Haloperidol administration attenuated the effects of MOD, resulting in a marked reduction in tail withdrawal, paw latency time, and withdrawal threshold by 55.3, 68.5, and 31%, respectively, when compared to the MOD group in the previously mentioned behavioral tests (Fig. 2).

### Modafinil Attenuated Depression and Enhanced Motor Coordination in Reserpine-induced Fibromyalgia in Rats

Compared to the normal group, RES significantly elevated immobility time in the FST by 2.3-fold and minimized rotarod-falling latency by 59.2%. The administration of MOD attenuated the action of RES, demonstrated by a 0.5-fold reduction in rat immobility time and a 1.2-fold increase in rat falling latency on rotarod relative to the RES group. On the contrary, HAL abated the beneficial effect of MOD by extending immobility time by 1.3-fold and reducing rat falling latency on rotarod by 61.8% as compared to the RES + MOD group (Fig. 2).

### Modafinil Alleviated Mast Cells-microglia Activation Cascades in the Thalamus of Reserpine-induced Fibromyalgia in Rats

**Fig. 3:**
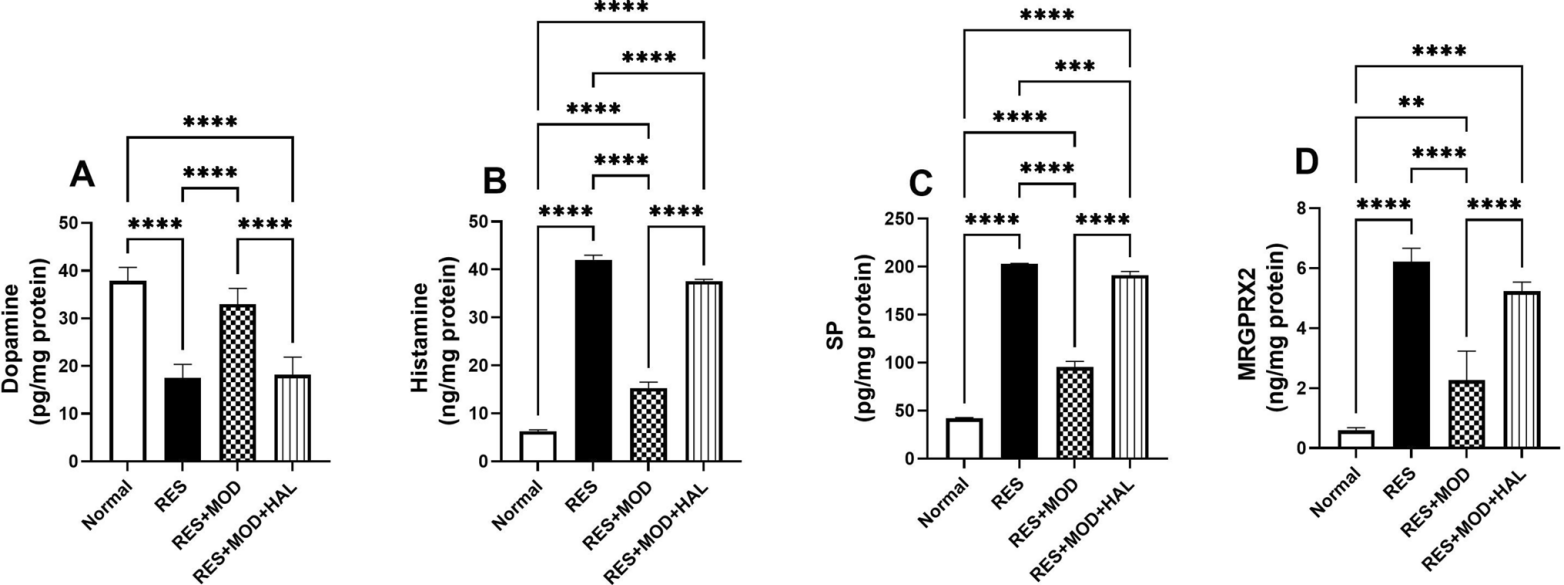
Effect of modafinil alone and combined with haloperidol on reserpine-induced alterations in thalamus content of dopamine (**A**), histamine (**B**), SP (**C**), and MRGPRX (**D**). Each bar with a vertical line represents mean ± SD (*n* = 5) using the one-way ANOVA test followed by Tukey’s as post hoc test for multiple comparisons post-test with a value *p < 0.05, **P < 0.01, ***P < 0.001, and ****P < 0.0001

As shown in Fig. 3, three consecutive daily injections of RES caused a significant depletion of DA in the rat thalamus by 54% compared with the normal group. Dopamine diminishment triggered an inflammatory response that turned on mast cell-microglial activation cascade through upregulation of SP, MRGPRX2, and histamine by 3.8-, 9.6-, and 5.7-fold, respectively, compared to normal rats, which resulted in degranulation of mast cells and the production of several inflammatory mediators in the thalamus. On the other hand, MOD abolished the effect of RES via boosting dopamine concentration in the thalamus by 88.5% and hampered the thalamic level of SP, MRGPRX2, and histamine by 52, 58, and 63.7%, respectively, compared to the FMS rats. However, HAL blocked the action of MOD and reduced the DA concentration by 44.8% compared to the treatment group. Furthermore, it increased the thalamic concentration of SP, the expression of MRGPRX2, and histamine level by 1-, 1.3-, and 1.5-fold, respectively, compared to the RES + MOD group.

### Modafinil Suppressed the Activation of PI3K/p-Akt Signaling in the Thalamus of Reserpine-induced Fibromyalgia in Rats

**Fig. 4:**
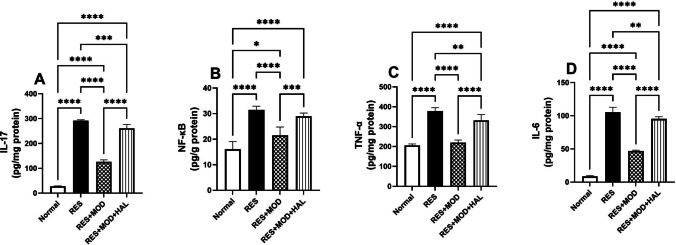
Effect of modafinil alone and combined with haloperidol on reserpine-induced alterations in thalamus content of PI3K (**A**), p-Akt/t-Akt (**B**), TNF-α (**C**), and IL-6 (**D**). Each bar with a vertical line represents mean ± SD (PI3K: *n* = 5, p-Akt/t-Akt: *n* = 3) using a one-way ANOVA test followed by Tukey’s as post hoc test for multiple comparisons post-test with a value *p < 0.05, **P < 0.01, ***P < 0.001, and ****P < 0.0001

Reserpine injection activated the PI3K/p-Akt pathway, which subsequently stimulated an inflammatory reaction in microglia. Compared to the normal group, diseased rats exhibited an increment in the thalamic levels of PI3K and p-Akt/T-Akt by 6.5- and 2.2-fold, respectively. However, MOD treatment resulted in a marked down-regulation in the thalamic levels of PI3K and p-Akt/t-Akt by 76.9 and 47.6%, respectively, compared to the RES group. Rats treated with HAL displayed an inhibitory effect on the MOD results of PI3K and p-Akt/T-Akt (Fig. 4).

### Modafinil Abridged the Inflammatory Mediators that Effectuated by Mast Cell Degranulation in the Thalamus of Reserpine-induced Fibromyalgia in Rats

**Fig. 5:**
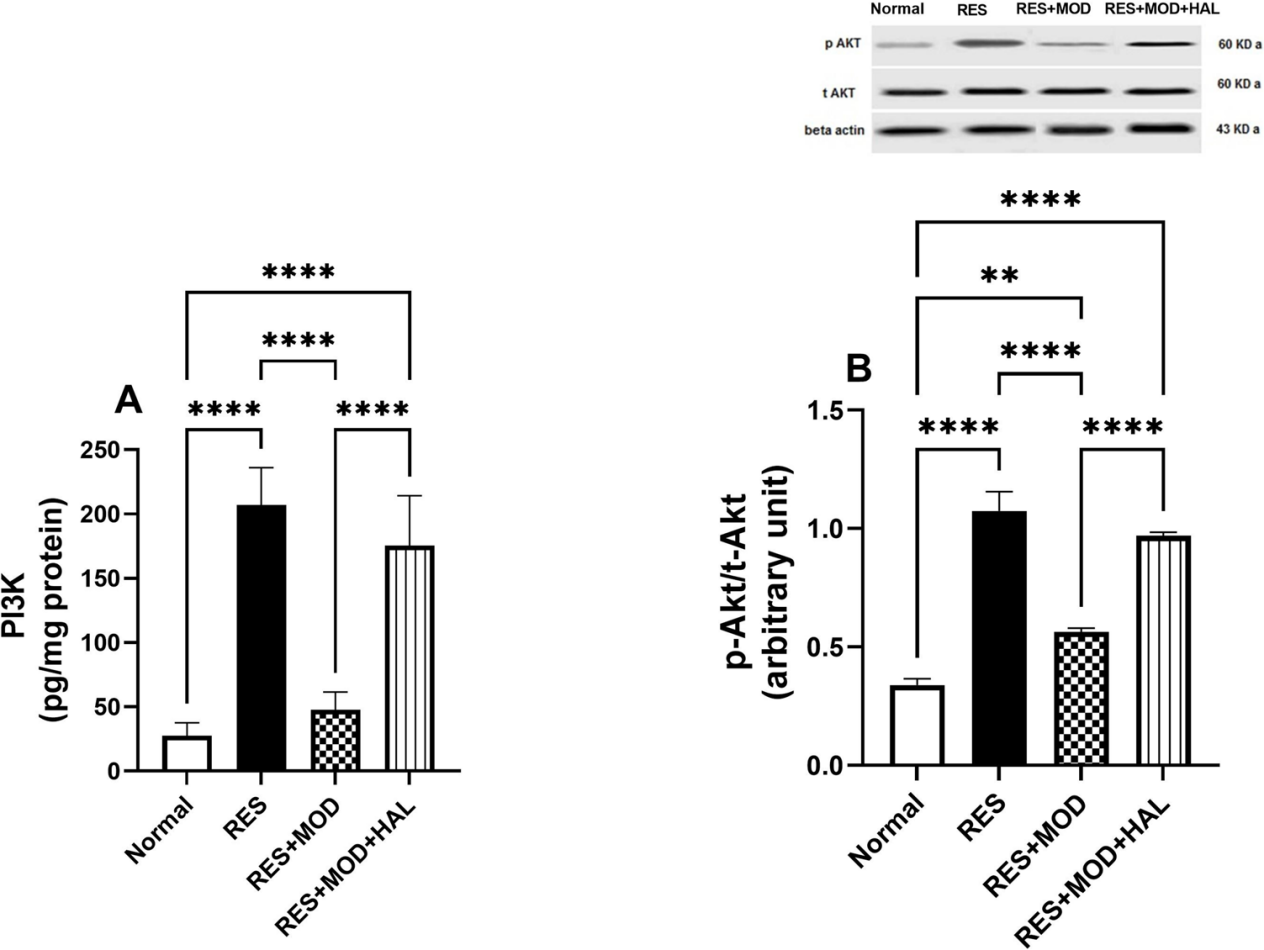
Effect of modafinil alone and combined with haloperidol on reserpine-induced alterations in thalamus content of IL-17 (**A**) and NF-κB (**B**). Each bar with a vertical line represents mean ± SD (*n* = 5) using a one-way ANOVA test followed by Tukey’s as a post hoc test for multiple comparisons post-test with a value *p < 0.05, **P < 0.01, ***P < 0.001, and ****P < 0.0001

A further consequence of RES-induced DA depletion is the increased production of IL-17 by 3.8-fold relative to the normal group, which partially facilitates the mast cell-microglial activation cascade, causing the upregulation of several thalamic proinflammatory cytokines, such as NF-κB, TNF-α, and IL-6, by 0.9-, 0.8-, and 11.2-fold, respectively, compared to the normal group. The immunomodulatory activity of MOD impaired the mast cell-microglial activation cycle and down-regulated the inflammatory mediators, IL-17, NF-κB, TNF-α, and IL-6, by 56.7, 31.7, 41, and 55.5%, respectively, in comparison to the RES group. Animals treated with HAL displayed a rise in the concentration of the inflammatory mediators of the thalamus (Fig. 5).

### Modafinil Restored the Histopathological Architecture Following Reserpine Administration in Fibromyalgia in Rats

#### H&E Staining

**Fig. 6:**
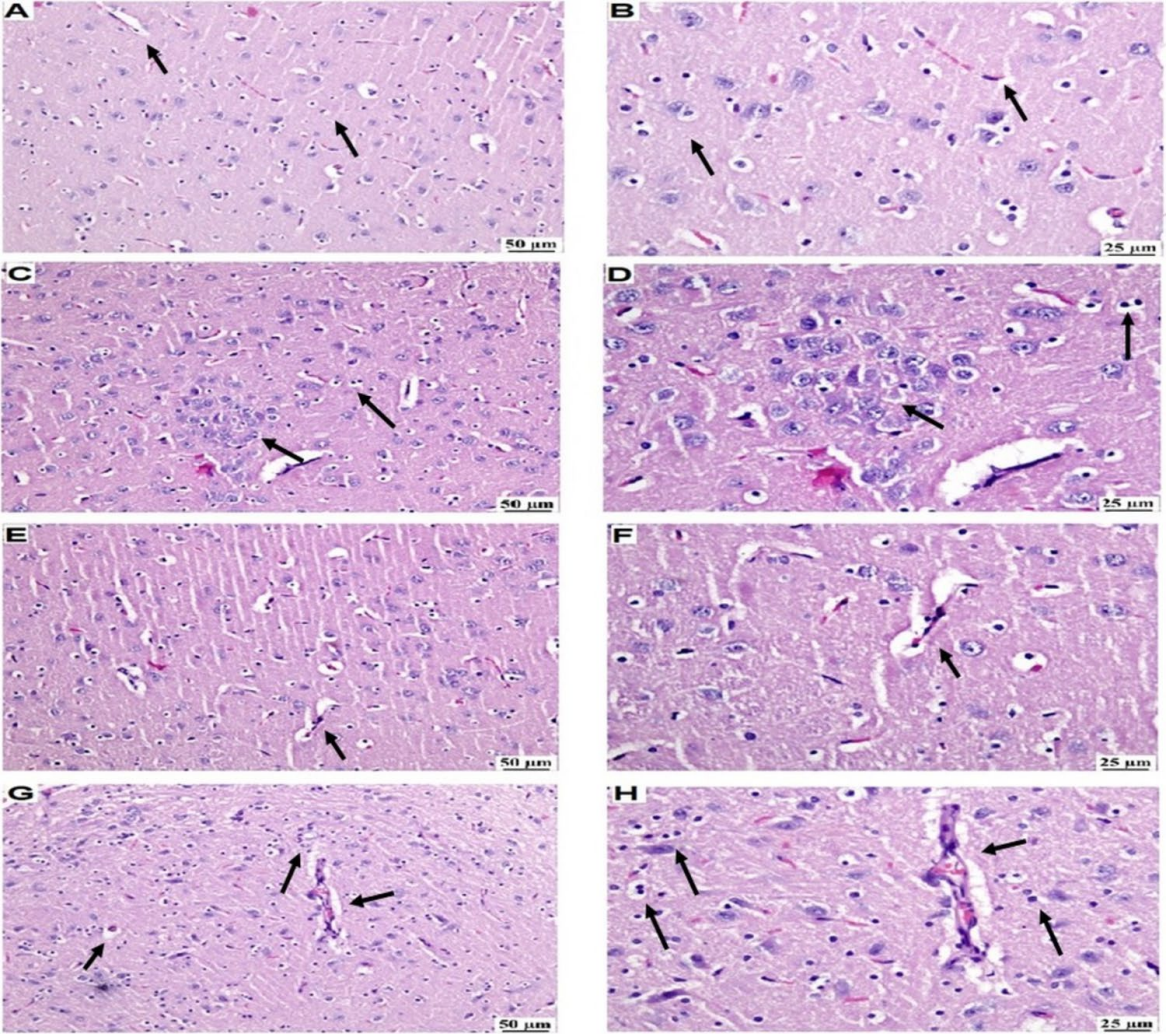
Photomicrographs of H & E-stained thalamus samples of all experimental groups (*n* = 3) (Scale bar: 50 μm, × 200, 25 µm, × 400) showing the effect of modafinil alone and combined with haloperidol on reserpine-induced histopathological alterations in thalamus. **A** and **B** normal group showing normal histology of thalamus. **C** and **D** RES group showing focal astrocytosis and diffuse gliosis. **E** and **F** the RES + MOD group showing normal thalamus. **G** and **H** the RES + MOD + HAL group displaying diffuse gliosis and thickened blood vessel walls

As shown in Fig. 6, no histopathological changes were detected in the thalamus of the examined brain sections from the normal group. Meanwhile, the thalamus of the RES group showed severely congested blood vessels. Numerous dark degenerating neurons with neuronophagia were observed. Additionally, the RES group's thalamus showed focal astrocytosis and diffuse gliosis. A marked improvement was observed in the MOD group, as the thalamus was normal which was characterized by more organized neuronal morphology. This included reduced neuronal degeneration, as evidenced by minimal signs of neuronal damage. Furthermore, there were minimal abnormal glial cell infiltration and astrocytes, indicating a lower level of neuroinflammation. The vasculature remained intact. However, HAL administration reversed the favorable effects of MOD, as manifested by numerous scattered dark neurons and diffuse gliosis with thickened wall blood vessels in the thalamus.

#### Toluidine Blue Staining

**Fig. 7:**
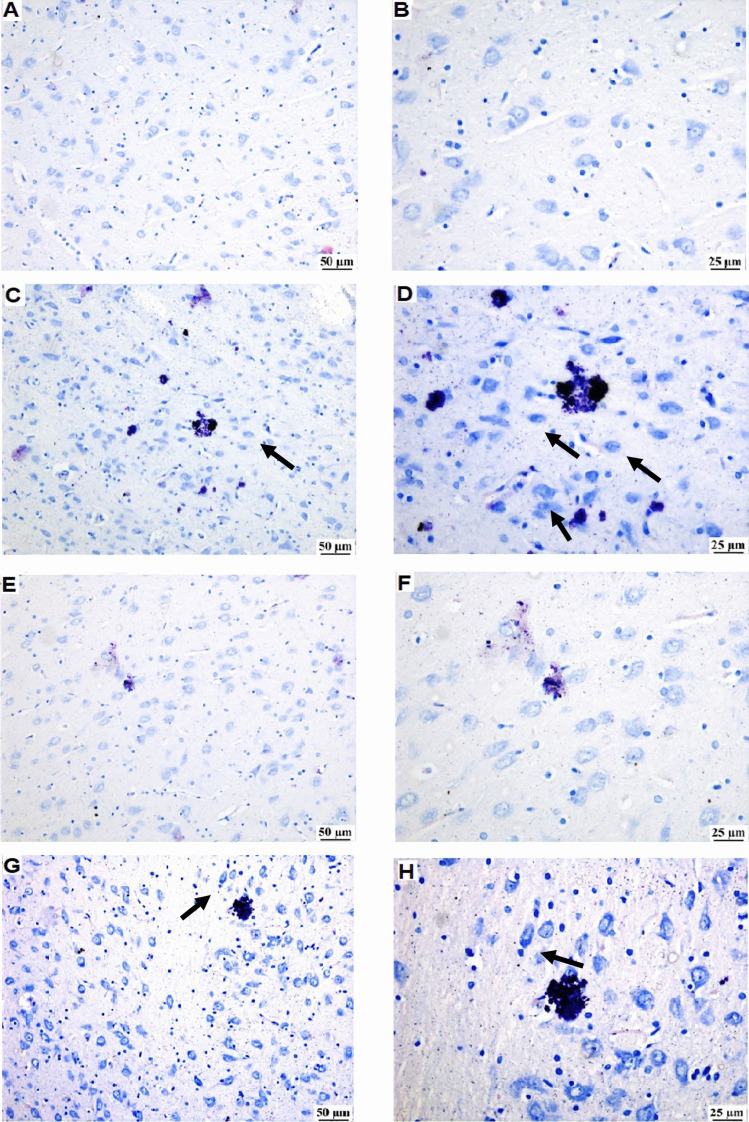
Photomicrographs of toluidine blue-stained thalamus samples of all experimental groups (*n* = 3) (Scale bar: 50 μm, × 200, 25 µm, × 400) showing the effect of modafinil alone and combined with haloperidol on reserpine-induced mast cell degranulation in the thalamus. **A** and **B** The normal group is showing normal faint stained neurons. **C** and **D** RES group showing numerous dark stained neurons (arrow). **E** and **F** The RES + MOD group is showing anormal thalamus. **G** and **H** the RES + MOD + HAL group is showing dark neurons (arrow)

Toluidine blue staining was used to confirm mast cell degranulation. Figure 7 illustrates that normal rats revealed faintly stained neurons. Meanwhile, the RES group exhibited numerous dark neurons. Treatment with MOD showed apparently normal neurons in the thalamus, while numerous dark neurons were observed after HAL administration.

### Modafinil Reduced Immunohistochemical Microglial Activation in the Thalamus Following Reserpine Administration in Fibromyalgia in Rats

**Fig. 8:**
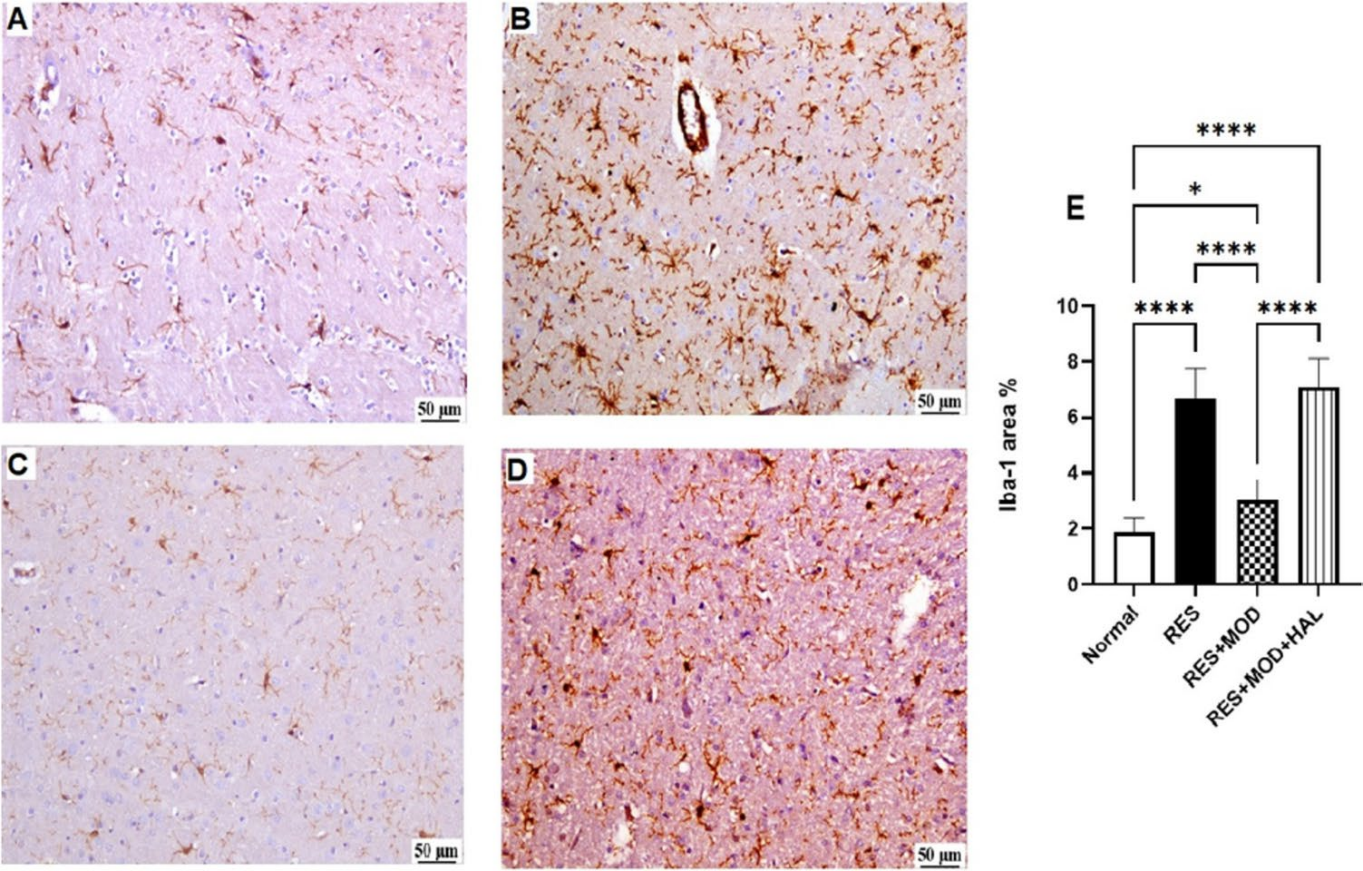
Photomicrographs of Iba-1 immunohistochemical staining of the thalamus of all experimental groups (*n* = 3) (Scale bar: 50 μm, × 200) showing the effect of modafinil alone and combined with haloperidol on reserpine-induced microglial activation. **A** Normal group showing limited expression of Iba-1 in the thalamus. **B** RES group showing higher expression of Iba-1 in the thalamus. **C** RES + MOD group showing lower expression of Iba-1 in the thalamus. **D** RES + MOD + HAL group showing higher expression of Iba-1 in the thalamus. **E** Iba-1-stained positive area % of the rats’ thalami. Each bar with a vertical line represents mean ± SD using a one-way ANOVA test followed by Tukey’s as a post hoc test for multiple comparisons with a value *p < 0.05, **P < 0.01, ***P < 0.001, and ****P < 0.0001

Iba-1 is specific microglia calcium-binding protein specifically expressed in microglia of rat brain cells, indicating the extent of cellular polarization. High thalamic expression of Iba-1 was evidenced after RES and HAL administration by 2.5- and 1.3-fold, respectively, when compared to the normal group. Meanwhile, lower expression was detected in the MOD group by 54.6% than that of the FMS group, indicating controlled microglial activation and subsequently reduced neuroinflammation (Fig. 8).

## Discussion

To the best of our knowledge, the current investigation is the first to reveal the efficacy of MOD in managing the common clinical aspects of fibromyalgia in the RES-induced FMS rat model. This finding is supported by the observed improvement in sensorimotor function, alleviation of depressive-like behavior, elevation of dopaminergic activity, attenuation of histopathological changes, and halting mast cell/microglia activation through curtailing SP/MRGPRX2/IL-17/histamine and PI3K/p-Akt/NF-κB signaling in the thalamus.

In the current study, FMS rats revealed a significant deterioration in cold immersion-tail withdrawal time, hot plate-paw withdrawal latency, and Randell-Selitto-paw withdrawal threshold. These changes reflected the occurrence of allodynia and hyperalgesia, which correspond with previous studies (Deuis et al. 2017; Atta et al. 2023a). Furthermore, diseased rats exhibited motor incoordination assessed by the rotarod test and a marked rise in immobility time in FST, indicating depression, as previously documented in several experimental (Nagakura 2022; Brum et al. 2022) and clinical studies (Yao et al. 2019). Widespread pain, allodynia, and hyperalgesia observed in FMS could be due to central sensitization (Mueller et al. 2021). Deficiencies in nociceptive transmission could disrupt pain input from every part of the body and strengthen the perception of pain to harmless stimuli (Woolf 2011). In the present study, MOD administration opposed the behavioral derangements of RES (allodynia and hyperalgesia) as manifested by the tail and paw withdrawal responses that were estimated using cold immersion and hot plate tests, respectively, besides increasing paw withdrawal threshold in the Randall-Selitto test. In accordance, Zager et al. (2018) displayed that a single MOD treatment can reduce the behavioral symptoms of an acute systemic inflammation caused by a high dosage of LPS. Modafinil also antagonized RES-induced depressive behavior assessed by FST and restored rotarod-tested motor coordination as previously reported (Omidi-Ardali et al. 2021; Katta et al. 2023).

It is mostly acknowledged that spinothalamic transmission of pain is the most significant ascending pathway for facilitating signals causing pain sensation (You et al. 2022). Hence, the thalamus plays a key role in pain processing. Dopamine is a significant neuromodulator associated with several neurological and psychiatric conditions (Takahashi et al. 2006). Dopamine-dependent effects are believed to have a prospective role in emotional and sophisticated somatosensory processing within thalamic circuitry (Takahashi et al. 2006). Any alteration in DA receptors in the thalamus may lead to several neurological and psychiatric disorders, as observed in FMS (Takahashi et al. 2006; Kane et al. 2009). Moreover, hypodopaminergic status and reduced availability of DA receptors have been linked to increased pain responses (Ledermann et al. 2016). In the present study, RES administration drastically diminished DA levels following former research, which revealed that the addition of RES to immune cell culture decreased the intracellular concentration of DA (Cosentino et al. 2000). Conversely, MOD increases extracellular DA levels by inhibiting its reuptake through binding to dopamine reuptake transporter (DAT) (Wisor 2013), an effect observed herein. Moreover, Zager (2020) suggested that MOD may directly influence DAT, which is constitutively expressed by glial and immunological cells (Zager 2020). Herein, the administration of MOD with HAL, a DA antagonist that specifically targets the D2 receptors (Fallon et al. 2019), was an approach to study the effect of MOD on dopaminergic signaling. Where administration of HAL reduced DA activity by blocking its receptor and increasing its turnover, an effect that counteracts the increase in DA signaling following MOD administration following previous work (Alam and Choudhary 2018). Consequently, HAL significantly reduced the observed beneficial effects of MOD on behavioral, biochemical, and histopathological changes induced by RES administration.

Interestingly, reduced DA levels may contribute to initiating neuroinflammation via stimulating mast cells-microglia activation cascades, ultimately increasing proinflammatory cytokines that are potentially implicated in the pathophysiology of FMS, as formerly explained (Skaper et al. 2017; Brum et al. 2024). Mast cell-induced neuroinflammation in the thalamus contributes to the progression of FMS (Skaper et al. 2017). The MRGPRX2 is one of the receptors that can trigger mast cells degranulation (Che et al. 2018), and its activation promotes inflammatory mechanical and thermal hyperalgesia (McNeil et al. 2015; Lu et al. 2019). Substance P is an immunomodulatory neurotransmitter released by nociceptive afferent nerve fibers as a consequence of stressful conditions, accompanied by a decline in DA levels, hence contributing to pain neurotransmission (Kempuraj et al. 2019; Belkacemi and Darmani 2020). Substance P activates MRGPRX2, causing degranulation of mast cells and initiating mast cells-microglia inflammatory cascades (Azimi and Lerner 2017; Varricchi et al. 2019). In the current study, the diminished thalamic DA content, observed after RES injection, led to an elevation of SP, which acted on the highly expressed MRGPRX2 receptors on the thalamic mast cells, triggering their degranulation as evidenced by the intense toluidine blue staining. Another factor implicated in mast cell activation and degranulation is IL-17, which acts on the IL-17A receptor expressed on the surface of the mast cells (Mills 2023). The reduction in DA levels stimulates IL-17 activity, leading to mast cell activation (Melnikov et al. 2016). This was reported in the current investigation as the thalamic IL-17 content was elevated after repeated RES administration, thus playing a role in mast cell degranulation (Theoharides et al. 2015). On the other hand, MOD halted the thalamic levels of SP and IL-17 and downregulated the expression of MRGPRX2, compared to the RES group. This resulted in the stabilization of thalamic mast cells, as supported by the low intensity of toluidine blue stain, according to Zager (2020). Similarly, MOD significantly reduced IL-17, IL-2, TNF-α, and NF-KB levels in the propionic acid rat model of autism‑like behavior (Bagcioglu et al. 2023).

Histamine, a mediator released upon mast cell degranulation, has been linked to arousal memory, depression, anxiety, and learning (Chikahisa et al. 2013). Histamine can induce microglial activation and migration via modulating PI3K/Akt/NF-κB signaling through its receptors on microglia, resulting in the secretion of several inflammatory cytokines including TNF-α, IL-1β, and IL-6 (Dong et al. 2014). Mast cell excitation/degranulation is responsible for nearly 90% of histamine content in the rat thalamus (Goldschmidt et al. 1985). Histamine is a prominent proinflammatory mediator and an immunological regulator of immune response (Mukai et al. 2018). The current findings revealed an increment in thalamic histamine levels accompanied by microglial activation, manifested by the high immunohistochemical expression of the Iba-1 stain, which enhances PI3K/p-Akt signaling in the thalamus of diseased rats. Accordingly, a prior study reported that the addition of histamine to the substantia nigra triggered microglial activation (Vizuete et al. 2000). In addition, microglia express DA receptors, particularly D2 receptors, which regulate neuroinflammation and survival levels (Dominguez-Meijide et al. 2017). Thus, microglial D2 receptor down-regulation or the reported reduction of DA in the current study may exacerbate neuroinflammation (Zhang et al. 2015). Herein, the immunomodulating effect of MOD is revealed via indirect modulation of histamine along with subsequent attenuation of microglia activation as manifested by low Iba-1 immune expression, as previously mentioned (Minzenberg and Carter 2008; Zager 2020). While MOD increases histamine release in the hypothalamus to promote wakefulness, its dopaminergic effects may simultaneously antagonize the histamine effect in peripheral and central immune cells through mast cell stabilization (Nakajima et al. 2020). The effect of MOD comprises a complex interplay between several neurotransmitters, including histamine, norepinephrine, and orexin. Modafinil enhances the release of norepinephrine and orexin, contributing to its wakefulness- and alertness-promoting properties (Minzenberg and Carter 2008; Battleday and Brem 2015). In addition to its conventional actions, orexin exhibited neuroprotective effects through its anti-inflammatory and immunoregulatory properties in many neurologic disorders with an immune component (Couvineau et al. 2019). Moreover, it has been reported that the neuroprotective activities of orexin are mediated by the PKC and PI3K signaling (Pasban-Aliabadi et al. 2017). Similarly, norepinephrine provides neuroprotection against several neurological and neurodegenerative disorders via abrogating oxidative stress and apoptosis, promoting autophagy, which reduces inflammation in neurons and glial cells and attenuates their activities (Ghasemi and Mehranfard 2024).

The activated microglia can be polarized to either traditionally activated M1 phenotype (proinflammatory) or activated M2 phenotype (anti-inflammatory). Latest studies showed that patients with nociceptive pain syndromes, such as FMS, have been shown to exhibit a disparity in the typical M1/M2 distribution that promotes systemic inflammation (Tripathi et al. 2021; Atta et al. 2023b). The polarization of activated microglia to M1 proinflammatory phenotype is triggered by the NF-κB signaling pathway (Zhang et al. 2013), which is responsible for the release of proinflammatory cytokines, such as TNF-α, IL-1β, and IL-6 (Safar et al. 2021; Elbadawy et al. 2025). In the current investigation, the activation of PI3K/p-Akt turned on NF-κB translocation into the nucleus that up-regulated the thalamic TNF-α and IL-6 contents. The release of inflammatory cytokines during microglial M1 activation is purported to induce cytotoxicity. This may lead to neurodegeneration of DA neurons, as evidenced herein by numerous dark degenerated neurons and neuronophagia, highly with severely congested blood vessels, focal astrocytosis, and diffuse gliosis.

On the other hand, MOD reduced microglial activation by inhibiting the PI3K/p-Akt/NF-κB pathway, resulting in lowering the pro-inflammatory cytokines TNF-α and IL-6 levels in the thalamus, as mentioned above (Cao et al. 2019; Zager 2020) and preserving the integrity of the neurons, as evidenced by restored histopathological architecture. In addition, D2 receptors are expressed on the surface of microglia, which regulates neuroinflammation. Thus, upon microglial D2 receptor stimulation by MOD administration, neuroinflammation can be controlled by decreasing NF-κB through an αβ-crystallin pathway (Zhang et al. 2015). Since the thalamic microglia play an essential part in amplifying pain sensation even in the absence of an initial painful stimulus (Vandenbark et al. 2019), halting mast cell/microglial activation by MOD treatment could restrain FMS and offering a ground for repurposing MOD for the management of FMS.

## Conclusion

The present study revealed MOD's efficacy in mitigating FMS symptoms in rats exposed to RES administration via increasing dopaminergic activity and curtailing SP/MRGPRX2/IL-17/histamine and PI3K/p-Akt/NF-κB signaling. The administration of MOD mitigated behavioral anomalies, histopathological disturbances, dopaminergic suppression, cytokines production, microglial activation, and mast cell degranulation. Taken together, MOD may have the potential to be repurposed in the management of FMS to enhance the patient's quality of life and productivity. Further experiments are essential to explore the effect of MOD using female and male rats to tackle the gender factor and ensure the generalizability of the findings. Also, it is essential to comprehensively elucidate the mechanisms of action underlying the therapeutic benefit of MOD in FMS, especially concerning dopamine receptor binding, downstream signaling, and the involvement of dopamine D2 receptors in microglial activation through the use of specific D2 agonists and antagonists.

## Data Availability

The datasets and materials generated during and/or analyzed during the current study are available from the corresponding author on reasonable request.

## Abbreviations

DA: Dopamine
FMS: Fibromyalgia syndrome
FST: Forced swimming test
HAL: Haloperidol
IL: Interleukins
MOD: Modafinil
MRGPRX2: Rat MAS Related GPR Family Member G
NF-κB: Nuclear factor-kappa B
p-Akt: Phosphorylated protein serine threonine kinase
PI3K: Phosphotylinosital 3 kinase
RES: Reserpine
SP: Substance P
TNF-α: Tumor Necrosis Factor Αlpha
